# The Relationship between Anxiety, Subjective and Objective Sleep, Chronotype and Circadian Rhythms with Depressive Symptoms in Insomnia Disorder

**DOI:** 10.3390/brainsci13040613

**Published:** 2023-04-04

**Authors:** Maria Comas, Alejandra Solis Flores, Nicole Lovato, Christopher B. Miller, Delwyn J. Bartlett, Ronald R. Grunstein, Julia Chapman, Christopher J. Gordon

**Affiliations:** 1 CIRUS, Centre for Sleep and Chronobiology, Woolcock Institute of Medical Research, 431 Glebe Point Road, Glebe, Sydney, NSW 2037, Australia; comassoberats@gmail.com (M.C.);; 2 Faculty of Medicine and Health, The University of Sydney, Science Rd., Camperdown, NSW 2050, Australia; 3 Renal Division, Department of Medicine, Faculty of Medicine and Medical Center, University of Freiburg, 79106 Freiburg, Germany; 4 Flinders Health and Medical Research Institute Sleep Health (Formally Adelaide Institute for Sleep Health: A Flinders Centre of Research Excellence), College of Medicine and Public Health, Flinders University of South Australia, Sturt Rd., Bedford Park, Adelaide, SA 5042, Australia; 5 Big Health Inc., 461 Bush St. #200, San Francisco, CA 94108, USA; 6 Sleep and Circadian Neuroscience Institute, Nuffield Department of Clinical Neurosciences, Level 6, West Wing, John Radcliffe Hospital, University of Oxford, Oxford OX3 9DU, UK; 7 CRC for Alertness, Safety and Productivity, Melbourne, VIC 3800, Australia; 8 Collaborative Centre for Cardiometabolic Health in Psychoses, Charles Perkins Centre-Royal Prince Alfred Hospital, Johns Hopkins Dr, Camperdown NSW 2006, Sydney Local Health District, Sydney, NSW 2050, Australia

**Keywords:** anxiety, chronotype, circadian preference, depressive symptoms, insomnia, melatonin

## Abstract

Insomnia is a highly prevalent sleep disorder with strong bidirectional associations with depressive symptoms. The circadian preference for eveningness has been shown to be associated with depressive symptoms in insomnia and other mental health conditions. However, there is a lack of studies in insomnia investigating whether objective measures, such as dim light melatonin onset (DLMO) or polysomnographic (PSG) sleep, are associated with depressive symptoms. Therefore, we investigated the associations between subjective measures (questionnaires assessing anxiety, sleep quality and circadian preference, and sleep diary) and depressive symptoms and whether the addition of objective measures (DLMO, PSG parameters) would strengthen the associations with depressive symptoms. In 115 insomnia disorder patients we found that anxiety was strongly associated with depressive symptoms in a model including circadian preference, dysfunctional beliefs of sleep, and self-reported previous depressive symptoms (R^2^ = 0.496, *p* < 0.001). The addition of sleep diary measures did not strengthen the model. We also found that the addition of objective measures (DLMO, PSG parameters) did not improve the subjective associations with depressive symptoms. Our data suggest that objective circadian markers are less important in the prediction of depressive symptoms in insomnia compared to subjective measures.

## 1. Introduction

Insomnia disorder is a highly prevalent sleep disorder affecting up to 10% of the adult population [1]. It impairs daytime functioning across cognitive, physical, social, and emotional domains, and can negatively impact work performance and increase accident risk rates [2,3]. Insomnia also has a strong bidirectional association with depression [4]. The psychopathology of depression is complex and multifactorial with genetic and environmental drivers involved in the development. However, insomnia can precede the onset of symptoms and coexist as a comorbid condition, and is often a key feature of the clinical symptoms of depression [5].

The human circadian system has a strong association with depressive symptoms [6]. The term chronotype has been used to describe either the preference for behaviour and /or sleep timing that may occur early (morning chronotype), late (evening chronotype), or in between the two (intermediate chronotype) in the 24 h day–night cycle [7]. Measurements of the chronotype vary from questionnaire-based tools such as Morningness-Eveningness Questionnaire (MEQ) and Composite Scale of Morningness (CSM) scales [7,8,9] which measure daily preference; sleep timing questionnaires such as the Munich ChronoType Questionnaire (MCTQ) which measures phase of entrainment [10]; or objective measurements, for example, the midpoint of the sleep period (the midpoint between sleep onset and offset) obtained from a sleep diary or activity monitoring [11]. Several studies have linked evening chronotype with different types of depression (e.g., major depressive disorder [12]) with a meta-analysis finding a small effect size (0.20) between eveningness chronotype and depressive symptoms [13]. This has been partly explained by linking evening chronotype with shorter sleep durations and worse sleep quality [14].

The most precise clinical measurement of the timing of the central circadian clock involves determining the onset of melatonin release in the evening under dim light conditions (dim light melatonin onset or DLMO) (see for example [15,16]). There is a reported strong relationship between DLMO and circadian preference determined by two different questionnaires [17], the MEQ [7], more linked to subjective preference, and the MCTQ [10,18] which uses self-reported sleep timing to estimate circadian preference. However, these studies examined the DLMO-circadian preference in normal healthy sleepers and Delay Sleep Phase Disorder samples. Previous research on DLMO and insomnia are limited, with one study finding later average DLMO times in insomnia compared to controls (22:02 h vs. 20:56 h, respectively [19]). Flynn-Evans et al. [19] found MEQ was associated with phase angle (bedtime-DLMO difference) in healthy-sleeping controls but not insomnia; however, it remains unclear how circadian preference and the timing of the central circadian clock is related in an insomnia population and how this relates to depressive symptoms.

Both evening circadian preference and insomnia are independently associated with non-remission of depression [20], and for anxiety and depressive symptoms in patients suffering from other severe mental illness [21]. Similarly, evening circadian preference and poor sleep quality are independently associated with depressive symptom severity in psychiatric inpatients with an affective disorder [22]. However, these studies have only evaluated the relationship between circadian preference and depression in insomnia patients using subjective measures of the circadian clock. There has been no study evaluating objective circadian markers. such as DLMO, and subjective markers of sleep-circadian behaviour in a clinical insomnia disorder population. This paucity of data exploring objective markers of sleep and circadian rhythmicity means there is a lack of understanding of why people with insomnia may be at greater risk of depressive symptoms. A better understanding of how objective sleep measures may predict depression or depressive symptomology in insomnia may enhance clinicians’ ability to screen for and identify depressive symptomology in their patients. Previous research about the depressive symptoms’ relationship in insomnia are exclusively based on subjective measures (questionnaires), and the evidence to date suggests that individuals with insomnia coupled with an eveningness chronotype are associated with depressive symptoms.

We therefore aimed to extend the existing literature by including objective measurements of sleep and circadian rhythm to assess the impact in patients with insomnia disorder. We hypothesized that associations between subjective measures (e.g., questionnaires) and depressive symptoms within an insomnia population would be improved with the addition of objective markers of sleep (PSG) and circadian rhythmicity (DLMO).

## 2. Materials and Methods

This study is from a secondary analysis of a larger insomnia phenotyping study conducted at the Woolcock Institute of Medical Research (WIMR), Sydney, Adelaide Institute of Sleep Health (AISH) and Flinders University, Adelaide. Briefly, participants were recruited from the community and clinics, and completed baseline questions prior to a telephone call and medical screening for insomnia disorder. Thereafter, participants completed two consecutive laboratory-based nights of overnight polysomnography with the second night measuring dim light melatonin onset (DLMO) testing with the participants allowed to sleep after completing testing (two-hour delay). The study was approved by Royal Prince Alfred Hospital Ethics Review Committee, Sydney, Australia (Protocol No X11-0392 & HREC/14/RPAH/517) and was prospectively registered with the Australian New Zealand Clinical Trial Registry (ANZCTR 12612000049875). All participants provided written informed consent prior to commencement of the study.

### 2.1. Participants

Participant eligibility was determined using the Insomnia Severity Index (ISI) score of ≥10 [23], coupled with a sleep interview and medical examination by an accredited sleep medicine practitioner. Participants needed to be diagnosed with insomnia disorder (DSM-5) which consisted of difficulty in initiating or maintaining sleep, waking early in the morning for at least three nights per week and continuous for at least three months, with daytime impairment [24]. They needed to be over 18 years, have English language fluency, and a bedtime habitually (as per sleep diary) before midnight. Participants were excluded based on the following criteria: self-reported pregnancy or lactating; active illicit substance usage; high alcohol (more than 14 standard drinks per week) or caffeine (>300 mg caffeine/day) usage; using medication likely to affect sleep, alertness, or melatonin; major psychiatric disorders; cognitive impairment; active shift-workers; recent trans-meridian travel (previous 2 months over 2 time zones); or actively treated sleep disorder.

### 2.2. Procedures

We recruited participants using an Internet-based website, through sleep clinics (WIMR, AISH and Flinders University), and via community advertisements. Potential participants completed sleep, general health, and lifestyle questionnaires prior to telephone screening. Following this, eligible participants were invited to attend the sleep clinics for consent and consultation for sleep assessment. Participants completed a 7-day sleep diary [25] prior to the laboratory visits. Participants then attended the laboratory for two consecutive nights, with overnight polysomnography (PSG) performed on night one and salivary DLMO collection on night two.

### 2.3. Assessments

#### 2.3.1. Polysomnographic Sleep Study

During night one of the laboratory visits, participants underwent overnight PSG with a bedtime between 22:00 and midnight. This consisted of electroencephalography (EEG) electrodes (F3, Fz, F4, C3, Cz, C4, Pz, O1, Oz, O2) with a ground electrode (FPz) and common reference (CPz), and electrooculographic and electromyographic (submental) recordings. The PSG signals were recorded using Embla (Mortara, Broomfield, CO, USA), Compumedics (Grael 4K, Charlotte, NC, USA) and (Somté PSG, Compumedics, Victoria, Australia) systems all at 512 Hz. Data were scored by an experienced sleep technologist according to American Academy of Sleep Medicine (AASM) criteria [26]. Sleep misperception was defined as: (objective (PSG) TST—subjective (sleep diary) TST/objective (PSG) TST) [27] with values ranging between −1 to +1, and positive values signifying an underestimation of objective TST and negative values overestimating objective sleep duration.

#### 2.3.2. Dim Light Melatonin Onset

Salivary melatonin was sampled hourly from 5 h before habitual bedtime to 2 h after with patients under direct or video observation for wakefulness and posture. All DLMO assessments were conducted between Tuesday and Friday. Habitual bedtime was determined from the sleep diaries and referred to the lights-out period and participants were attempting sleep. Participants maintained their usual sleep–wake schedule in the week prior to the laboratory visits. Saliva samples were collected in dim light conditions (<10 lux), with the participants in an upright seated posture for at least 20 min prior to sampling. Samples were stored immediately in a −20 °C freezer until later laboratory analysis. Salivary melatonin concentrations were determined by a radioimmunoassay method (Buhlmann Laboratories; Allschwil, Switzerland). These assays have a limit of detection of 1 pg/mL with an inter-assay coefficient of variation of 7.4% at 4.41 pg/mL and 10.7% at 48.14 pg/mL. DLMO was calculated using linear interpolation with a melatonin concentration threshold of 3 pg/mL [28]. Phase angle was determined from the difference in average self-reported bedtime based on a 7-day sleep diary (as phase marker for the synchronizing cycle, i.e., light offset) and DLMO time (as a circadian phase marker).

#### 2.3.3. Questionnaires

A series of questionnaires was completed at baseline. Insomnia Severity Index (ISI), a 7-item questionnaire, was used to assess insomnia symptom severity [29]. Depressive symptoms were assessed using the Patient Health Questionnaire (PHQ-9), a 9-item validated questionnaire with strong psychometric properties [30]. Cut-off scores for PHQ-9 are <4 no depressive symptoms, 5–9 mild, 10–14 moderate, 15–19 moderately severe, and 20–27 severe depressive symptoms. The 16-item version of the Dysfunctional Beliefs and Attitudes About Sleep scale (DBAS-16) [31] was used to evaluate the role of sleep-related beliefs and attitudes. The General Anxiety Disorders (GAD-7) [32], a 7-item screening tool, was used to assess anxiety levels. Circadian preference was assessed using the 13-item Composite Scale of Morningness (CSM) questionnaire [8]. The CSM is a reduced version of the Horne and Östberg Morningness–Eveningness questionnaire (MEQ; [7] to assess circadian preference. Cut-off scores for CMS are ≤22 evening type, 23–44 intermediate, ≥45 morning type. The Pittsburgh Sleep Quality Index (PSQI) [33] was used to assess subjective sleep quality. The cut-off for PSQI is >5 poor quality sleep.

### 2.4. Data Analysis

All data are reported as means and standard deviations (SD) unless otherwise specified. Data were checked for normality using visual inspection of Q-Q plots. Associations between DLMO and circadian preference were assessed using Pearson’s correlation. Associations between depressive symptoms (PHQ-9) and the independent variables of anxiety, circadian preference, dysfunctional beliefs, subjective sleep quality, sleep misperception, objective and subjective sleep timings, DLMO, and phase angle were determined using linear multiple regression. To avoid multicollinearity, variables in each model were first chosen based on their individual correlation with depressive symptoms (i.e., a single measure from the sleep diary was selected and a single sleep quality measure from PSG was selected). Linear regression models were first completed utilizing all variables of interest then repeated using stepwise regression to statistically remove variables that added nothing further to the model. We undertook the following approach to the linear regression models: (i) studies that used basic, clinical variables that may be collected within a sleep consultation including questionnaires and the sleep diary; (ii) we included objective measures (DLMO, phase angle, PSG metrics) collected within a sleep laboratory setting. Confounders (age, sex and BMI) were controlled for across all regression analyses. The DLMO cut-off of 22:00 h was based on [34]. As sleep disordered breathing may have influenced the analyses, we conducted sensitivity analyses by removing participants with an Apnea-Hypopnea Index (AHI) ≥15 events/h to ascertain the effect on the models. We deliberately did not apply correction factors as we were interested in examining associations that may have arisen from these analyses. Alpha was set at <0.05 for all analyses. Data were analysed using SPSS software (IBM v 25.0.0; IBM Corp, Armonk, NY, USA).

## 3. Results

**Figure 1:**
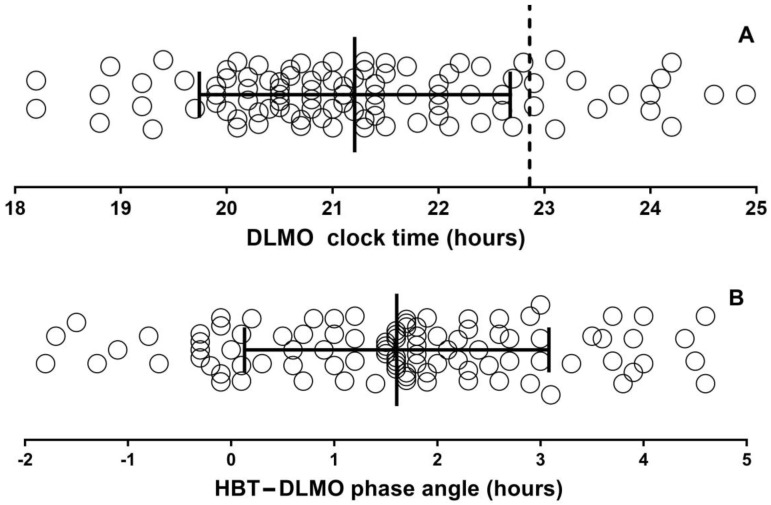

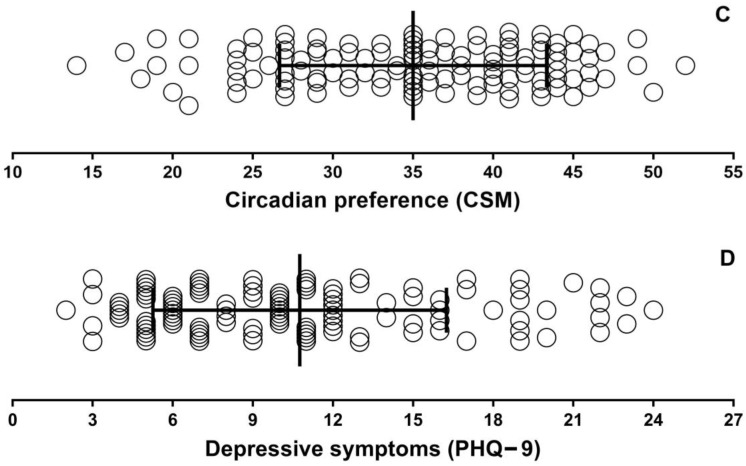
Dim light melatonin onset clock time (**A**), phase angle (**B**), circadian preference (Composite Score of Morningness) (**C**), and depressive symptoms (**D**) for all participants. Vertical dashed line in A shows average habitual bedtime (diary). Solid lines represent mean and SD. Key: DLMO—dim light melatonin onset; HBT—habitual bedtime sleep; CSM—Composite Scale of Morningness; PHQ-9—Patient Health Questionnaire.

A total of 115 participants were included in the study. DLMO was determined in 98 participants (did not reach threshold n = 8, insufficient saliva n = 3, DLMO not recorded n = 6). Demographics are provided in Table 1, which shows they were predominantly female (70%) and middle-aged (47.1 years ±14.8, range 18–80). There was no difference in age, BMI, or ISI, CSM, PSQI, GAD7, or PHQ9 scores between those who performed DLMO testing and those who did not. The average DLMO was 21:12 (SD 1:28) with an average sleep diary habitual bedtime clock time of 22:52 (SD 0:56) (Figure 1A) and average phase angle (habitual bedtime—DLMO) of 1:36 (SD 1:29) (Figure 1B). A total of 16.3% of participants had a DLMO after their HBT_d_. The average score for CSM was 35, with most participants having intermediate 80.0%, 12.2% morning, and 7.8% evening circadian preference (Figure 1C). Self-reported episodes of previous anxiety and depressive symptoms occurred in 20.9% and 31.3% of the cohort, respectively. There was a range of PHQ-9 depressive symptom scores (overall 10.8 (SD 5.5) range: 2–24): no depressive symptoms n = 10 (8.7%); mild n = 42 (36.5%); moderate n = 35 (30.4%); moderately severe n = 18 (15.7%); severe n = 10 (8.7%) (Figure 1D). Thirty-two (27.8%) and sixteen (13.9%) participants had taken sleep medication (benzodiazepines, z-drugs, melatonin) and anti-depressants (SSRI, SNRI) within the last six months.

**Figure 2:**
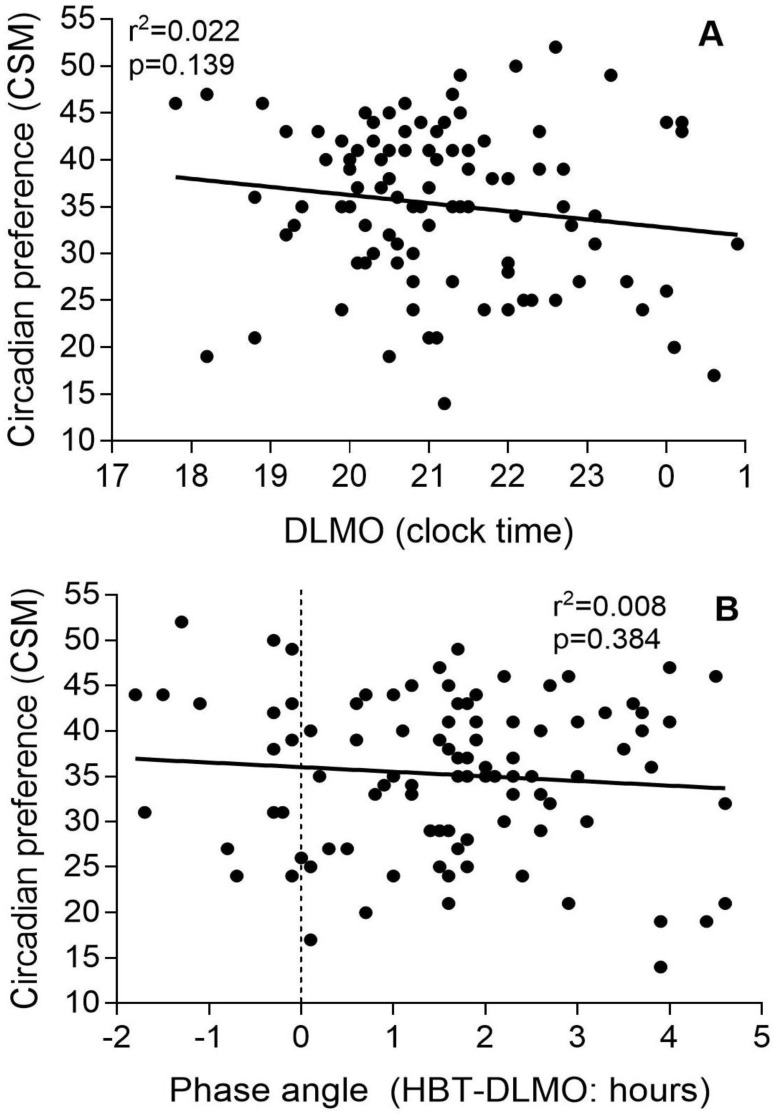
Association between dim light melatonin onset and circadian preference (Composite Score of Morningness) (**A**), and phase angle (habitual bedtime—DLMO) and (**B**) circadian preference (Composite Score of Morningness).

**Figure 3:**
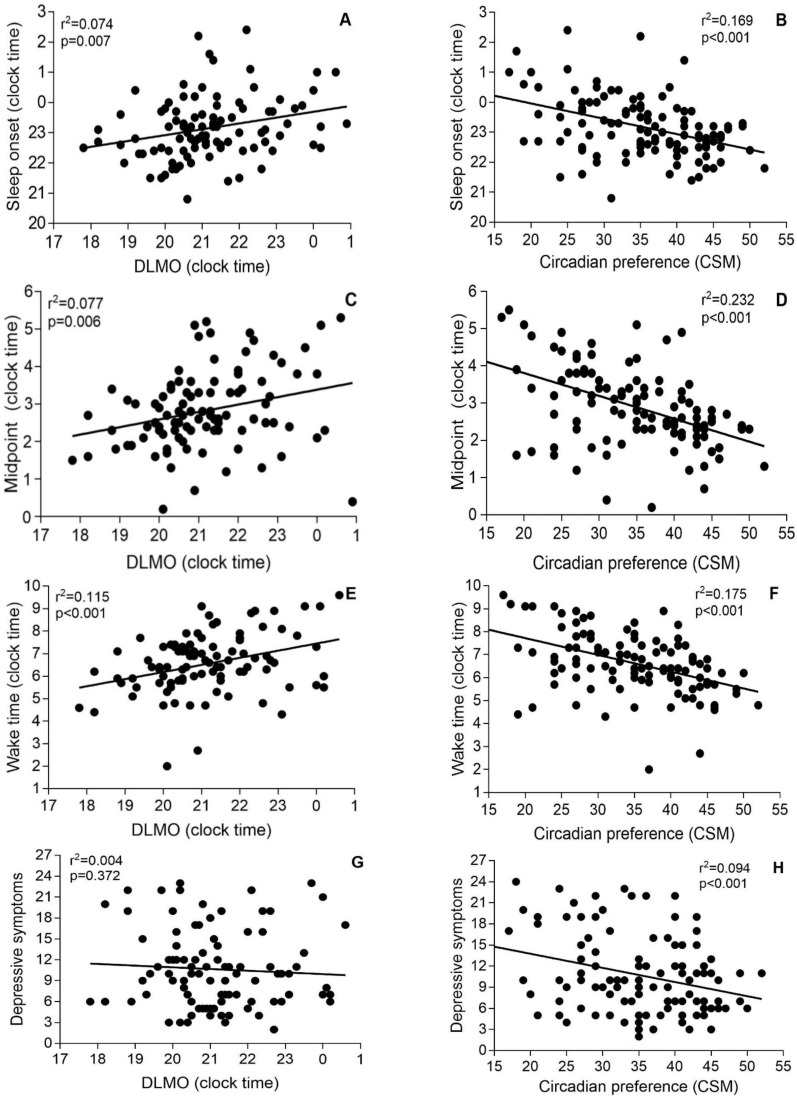
Associations between dim light melatonin onset and sleep-diary-derived sleep onset time (**A**), sleep midpoint (**C**), wake time (**E**), and depressive symptoms (**G**); and associations between chronotype and sleep onset time (**B**), sleep midpoint (**D**), wake time (**F**), and depressive symptoms (**H**).

There were no significant correlations between DLMO and phase angle with circadian preference (Figure 2). However, there were correlations between DLMO and sleep onset (R^2^ = 0.074, *p* = 0.007), midpoint (R^2^ = 0.077, *p* = 0.006), and wake time (R^2^ = 0.115, *p* < 0.001), as well as between circadian preference and sleep onset (R^2^ = 0.169, *p* < 0.001), midpoint (R^2^ = 0.232, *p* < 0.001), and wake-up time (R^2^ = 0.175, *p* < 0.001) (Figure 3). DLMO was not correlated with PHQ-9 scores (Figure 3G), but CSM scores did correlate with PHQ-9 (Figure 3H).

The same correlations were calculated after dividing participants with early and late DLMOs using 22:00 as the cut-off (Table 2). The correlations between DLMO and sleep onset time or DLMO and depressive symptoms were not significant in both groups. For midsleep and wake-up times, only the correlations with participants with DLMO before 22:00 were significant.

Correlations between subjective and objective variables are presented in Table S1. There were significant correlations between depressive symptoms (PHQ-9) and anxiety (GAD-7), sleep quality (PSQI), circadian preference (CSM), and dysfunctional beliefs about sleep (DBAS)). Correlations existed between sleep quality and DBAS (*p* = 0.007); anxiety (GAD-7; *p* < 0.001); total sleep time_diary_ (*p* < 0.001); and sleep onset latency_diary_ (*p* < 0.001). Overall, the cohort underestimated sleep (misperception index: −0.115, SD 0.294, range: −1.0–0.55). There was no statistical association with depressive symptoms, although the sleep misperception index was correlated with sleep quality and sleep diary metrics (Table S1).

After correcting for known confounders, multiple regression was used to determine associations between depressive symptoms (PHQ-9) and subjective variables (anxiety, circadian preference, dysfunctional beliefs of sleep previous depressive symptoms), revealing statistically significant associations (Table 3; *p* < 0.001). The addition of sleep diary measures did not strengthen the association, nor did the addition of objective measures (DLMO, PSG).

Anxiety was consistently strongly associated across all models and was independently associated with depressive symptoms (β = 0.685, 95%CI 0.517–0.852, *p* < 0.001). TST_PSG_ and WASO_PSG_ were independently associated with depressive symptoms (*p* = 0.008), but their addition alongside the subjective measures did not improve the prediction of depressive symptoms.

There were 16 participants with an AHI ≥ 15 (32.9 vs. 4.5 events/h) with baseline characteristics similar to the overall cohort, except for WASO (PSG) (80.6 vs. 118.8 min; *p* = 0.017). When controlling for an AHI ≥ 15, we found no change in the associations between subjective and objective variables and depressive symptoms across all models.

## 4. Discussion

We investigated a community-based, clinically diagnosed insomnia population to examine associations between depressive symptoms and a comprehensive set of subjective and objective sleep and circadian measures. The main finding from our study was that subjective measures that could be used in clinical practice were strongly predictive of depressive symptoms in insomnia disorder. Whilst we also found that some objective measures (PSG TST, and WASO) were predictive of depressive symptoms in insomnia, when combined with the subjective measures these did not improve the prediction of depressive symptoms. We also found that evening circadian preference, but not DLMO and phase angle, was correlated with depressive symptoms. Interestingly, we observed no association between circadian preference and DLMO or phase angle in this patient sample. This suggests an interplay of subjective and objective sleep and circadian clock variables differentially affecting depressive symptoms in those with insomnia.

In accordance with previously published studies [35], anxiety was strongly associated with depressive symptoms in our study.

Anxiety and insomnia often coexist and recent evidence shows that treating insomnia disorder using cognitive behavioural therapy improves anxiety to a level comparative to anxiety-specific therapy [36]. Our findings support other research that anxiety is closely related to depression in insomnia, with a relationship that is not further explained by circadian markers [37].

Several reports have shown that DLMO significantly correlates with circadian preference (MEQ) in healthy participants [38,39], epilepsy patients [39], and in depressed and non-depressed peri- and post-menopausal women [40]. In contrast, correlations were not found in healthy subjects nor in participants with traumatic brain injury (TBI) [41]. Two studies compared the correlations between DLMO and circadian preference assessed by MEQ and MCTQ [17,42]. Kantermann et al. [17] merged the data of both healthy and Delayed Sleep–Wake Phase Syndrome (DSWPD) subjects for their correlations of DLMO with circadian preference and, regardless of the method used to assess circadian preference, they found strong and highly significant correlations [17]. Post-hoc analysis of the same data showed that correlations between DLMO and mid-sleep for free and working days determined by MCTQ were stronger in males than in females [16]. Conversely, Kitamura et al. [42] found a significant correlation between DLMO and various MCTQ parameters but not between DLMO and MEQ in healthy participants. As for insomnia, previous research has not found a correlation between circadian preference (MEQ) with phase angle (bedtime-DLMO) in an insomnia cohort but found correlations in their control group [19]. In the present study and that of Flynn-Evans et al. [19], there were no correlations between circadian preference and DLMO or phase angle in the insomnia populations. The reasons for this are unclear. Previous research has shown that sleep phase markers (bedtime, midpoint, and wake-up time) determined by sleep diaries or logs are significantly associated with DLMO [38,43]. Moreover, it has been shown that a change in sleep timing, i.e., delaying wake-up time, has a profound effect on both dim light melatonin onset and offset times, delaying both by about 2.5 h [44]. Interestingly, Reiter et al. [45] investigated which phase markers derived from the Pittsburgh Sleep Quality Index, Munich Chronotype Questionnaire, sleep diaries, and actigraphy were best for estimating DLMO in healthy subjects, and found that sleep midpoints from sleep diaries or actigraphy were the best estimates of DLMO. It is considered that circadian preference assessed using MEQ or CSM questionnaires measures a trait and not a state of the individual. This may be even more pronounced in people with insomnia; hence, the lack of correlation between the measure of trait-like (circadian preference determined by questionnaire) and state-like (DLMO) characteristics of insomnia disorder.

Insomnia is a highly heterogeneous disorder with different sub-types, including those where insomnia is co-morbid with another disorder or, in some instances, where insomnia may be the symptom of another disorder rather than the disorder per se [46,47]. Interestingly, some insomnia sub-types may have altered circadian timing and chronotypes, which has been suggested as a risk factor for insomnia [48,49,50]. Lack and colleagues (1996) showed that insomnia patients who exhibited early morning awakening had significantly earlier DLMO times (20:30 h [SD 1:05]) compared with controls (average of the difference with control DLMO 2:15) [50]. Conversely, Flynn-Evans et al. [19] reported that insomnia patients in their cohort had later DLMO times (22:02, SD 2:02) compared to the controls (average of the difference with control DLMO 2:06) but with no correlation between sleep onset and phase angle. In our study, average DLMO occurred at 21:12 (SD 1:28), and circadian preference was more strongly correlated than DLMO with the three sleep phase markers, i.e., sleep onset, midpoint, and end of sleep but all the correlations were significant (Figure 3). Wright et al. [34] stratified insomnia patients into early and delayed DLMO subgroups (using DLMO at 22:00 as cut off) and found correlations between DLMO and sleep onset time, sleep midpoint and wake-up time were only evident in the delayed subgroup [34]. In contrast, we did not find any significant correlations between DLMO and the sleep phase markers for the delayed DLMO subgroup but significant correlations between the early DLMO group and both sleep midpoint and wake-up time (Table 2). It has been shown in previous research that midsleep and wake-up time are more strongly correlated with DLMO than bedtime [38]. The discrepancy between our results and those of Wright et al. [34] may be explained by the high insomnia heterogeneity and underlying circadian disorders in some of the insomnia participants. Our study population was limited to those with a habitual bedtime before midnight, unlike others that allowed a much later bedtime [19], restricting recruitment of patients with severe delayed circadian preference and who are thus more likely to have circadian rhythm disorders.

In the insomnia population, morningness–eveningness preference might not relate in the same way to the circadian system, using DLMO as a proxy, compared to other populations due to the pathophysiology of the disorder. The participants’ daily behaviour, light exposure, or social interactions may also be impacted in insomnia, and these external factors impact the circadian system (assessed using DLMO). If that is the case, it may be that the time preferences in their behaviour (trait-like rather than state-like characteristics) remain more closely related to their circadian system and its relationship with depression. This would also partly explain why circadian preference, assessed by questionnaire, predicts depressive symptoms but not DLMO in our cohort. The determination of circadian preference may vary depending on what assessment tool is used. In our case, we used a composite score of morningness scale which may relate poorly to objective circadian measures compared to questions such as the MCTQ [10]. However, the CSM and MEQ are widely used and have been satisfactorily validated by different authors [51]. Our results show that circadian preference but not DLMO predicts depressive symptoms, which suggests that unless an underlying circadian rhythm disorder is suspected, objective melatonin measurement in insomnia disorder does not provide useful diagnostic information. Interestingly, several studies have also found a lack of correlation between subjective and objective measures of sleep, and that those subjective, rather than objective, sleep measures better predicted depressive symptoms in their cohorts [52,53]. Thus, it is worth considering the possibility that perception and preference, rather than actual objective sleep and circadian clock measures, better relate to depressive symptoms. More work is needed to understand and interpret the discrepancy between subjective and objective measures in the insomnia population. However, in our view, it would not be useful or be cost effective to undertake routine DLMO testing in the diagnosis of insomnia.

## 5. Strengths and Limitations

This is the first study to examine a comprehensive set of objective and subjective variables from a community-dwelling insomnia population. This study included the largest number of DLMO assessments, PSG, and a battery of self-reported questionnaires in insomnia patients and showed a lack of association between DLMO and depressive symptoms in insomnia. It should be noted that we only included patients with a habitual bedtime of earlier than midnight, meaning that we excluded those with extreme habitual bedtimes. These extreme bedtimes could be associated with an underlying circadian disorder, and our aim was to evaluate a clinical insomnia population. This has clinical implications as it strongly suggests that assessing DLMO does not confer clinical benefit in insomnia unless an underlying circadian disorder is suspected.

There are several limitations in this study. First it was a cross-sectional study that cannot be used to infer causality due to the lack of temporality. Furthermore, despite a well-classified sample with objective and subjective variables, the lack of repeated measures precludes direct relationships between predictors and outcome. Second, we did not have a wide range of circadian preferences, with no participants in the extreme range, even though there was a large range of melatonin onset times. This was likely due to excluding participants with a habitual bedtime after midnight. Third, the use of a questionnaire to assess depressive symptoms is another limitation and participants were not directly clinically assessed for depression or depressive symptoms. Fourth, we conducted an overnight sleep study using PSG on one night only and the insomnia participants may have experienced a reverse first night effect resulting in improved sleep metrics. Fifth, it would have been helpful to compare our insomnia participants to healthy sleepers to determine if the same results were found. Further research should incorporate age, and sex-matched cases and controls. Finally, our sample size was modest but larger than the only previously published study that examined DLMO and chronotype in insomnia (n = 68; [19]). Nonetheless, this may explain the lack of associations between different parameters and depressive symptoms or between subjective and objective measures of chronotype.

## 6. Conclusions

There were several subjective and objective variables that predicted depressive symptoms in these insomnia patients. Although anxiety was the strongest correlate of depressive symptoms in insomnia, we found associations between depressive symptoms with DBAS, previous depressive symptoms, TST_PSG_, and subjective, but not with objective, circadian measures. These data suggest that clinicians need to focus on reducing anxiety. Further, the relationship between depressive symptoms and insomnia-related objective and subjective measures, including circadian measures, requires assessment using longitudinal studies to determine the temporal nature of insomnia and depressive symptoms. Importantly, the present study has shown that the assessment of circadian preference as determined by the CSM questionnaire was useful for predicting depressive symptoms in the insomnia population. Therefore, future studies could aim to systematically investigate whether assessment of circadian preference, e.g., MEQ, CSM, or entrainment phase using MCTQ, better predicts depressive symptoms in different population types, including insomnia.

## Figures and Tables

**Table 1:** Demographic, sleep and clinical measures of the participants. Key: ISI—Insomnia severity index; PSQI—Pittsburgh sleep quality index; CSM—Composite Scale of Morningness; DBAS—Dysfunctional beliefs about sleep; GAD-7—Generalised Anxiety Disorder; PHQ-9—Patient Health Questionnaire; DLMO—dim light melatonin onset; PSG—polysomnography; TST—total sleep time; SOL—sleep onset latency; WASO—wake after sleep onset; HBT_d_—habitual bedtime sleep diary. Note: one participant recorded zero minutes on the PSG recording which was not recorded in the ranges.

Variable	Mean ± SD (Range) n = 115
Age (years): SD	47.1 ± 14.8 (18–80)
BMI (kg/m^2^)	25.6 ± 5.3 (17.1–43.8)
Female: n (%)	80 (70%)
ISI	19.9 ± 4.2 (10–28)
PSQI	13.1 ± 3.3 (5–20)
CSM	35.0 ± 8.3 (14–52)
DBAS	5.7 ± 1.6 (1–9)
GAD-7	7.2 ± 4.9 (0–21)
PHQ-9	10.8 ± 5.5 (2–24)
DLMO (hh:mm) (n = 98)	21:12 ± 1:28 (17:28–0:32)
PSG: TST (min)	344.2 ± 77.1 (156.0–518.5)
PSG: SOL (min)	22.0 ± 26.4 (1.0–159.0)
PSG: WASO (min)	86.0 ± 54.4 (7.0–249.5)
PSG: sleep efficiency (%)	74.1 ± 14.4 (43.5–94.4)
Diary: TST (min)	346.6 ± 87.2 (135.0–557.1)
Diary: SOL (min)	43.0 ± 39.0 (5.6–180.0)
Diary: WASO (min)	57.6 ± 50.0 (2–252.0)
Diary: sleep efficiency (%)	75.7 ± 15.2 (39.1–98.2)
Phase angle HBT_d_—DLMO (min)	96.3 ± 88.6 (−108–276)

**Table 2:** Correlations between subgroups of participants depending on their DLMO times, before and after 22:00 with sleep diary variables (onset, midsleep, and wake-up time) and depressive symptoms. DLMOs, sleep variables, and depressive symptoms are shown as averages (SD) and (range). The symbol * indicates statistical significance.

Insomnia Subgroups	DLMO	Sleep Onset Time	r *p* Values	Midsleep	r *p* Values	Wake-Up Time	r *p* Values	Depressive Symptoms	r *p* Values
DLMO <22:00 (n = 69)	20:30 (0:54) (17:48–21:48)	23:00 (1:00) (20:48–2:12)	0.196 *p* = 0.107	2:42 (0:54) (0:12–5:12)	0.276 *p* = 0.022 *	6:18 (1:12) (2:00–9:06)	0.256 *p* = 0.034 *	10.6 (5.4) (3–23)	−0.225 *p* = 0.063
DLMO ≥22:00 (n = 29)	23:00 (0:54) (22:00–0:54)	1:30 (1:06) (21:30–2:24)	0.097 *p* = 0.616	3:12 (1:12) (0:24–5:18)	−0.097 *p* = 0.943	6:54 (1:42) (1:36–9:36)	−0.205 *p* = 0.286	10.7 (5.9) (2–23)	0.089 *p* = 0.645

**Table 3:** Multiple regression analyses of associations with depressive symptoms in insomnia. Key: Circadian preference—CSM; Dysfunctional beliefs—DBAS; Anxiety—GAD-7; TST_diary_—total sleep time from diary; TST_PSG_—polysomnographic total sleep time; DLMO—dim light melatonin onset.

Predictors	Model 1				Model 2				Model 3			
	B	SE	β	*p*	B	SE	β	*p*	B	SE	β	*p*
Anxiety	0.565	0.085	0.495	**<0.001**	0.508	0.091	0.461	**<0.001**	0.491	0.092	0.445	**<0.001**
Circadian preference	−0.072	0.056	−0.110	0.202	−0.075	0.058	−0.124	0.196	−0.066	0.059	−0.109	0.265
Dysfunctional beliefs	0.425	0.264	0.120	0.111	0.334	0.272	0.102	0.222	0.368	0.276	0.112	0.185
Previous depression	2.073	0.935	0.174	**0.029**	1.804	0.96	0.161	0.63	1.865	0.951	0.166	0.053
TST_diary_					−0.43	0.299	−0.012	0.887	0.047	0.305	0.013	0.879
TST_PSG_									−0.011	0.005	−0.165	**0.049**
DLMO									0.001	0.001	0.058	0.457
R^2^	**0.529**		**Adjusted 0.496**		**0.475**		**Adjusted 0.430**		**0.508**		**Adjusted 0.453**	

## Data Availability

The datasets generated and/or analysed during the current study are not publicly available but are available from the corresponding author on reasonable request.

## Supplementary Materials

The following supporting information can be downloaded at: https://www.mdpi.com/article/10.3390/brainsci13040613/s1, Table S1: Correlations between subjective and objective variables.
