# Supplementary material for: The Relationship between Anxiety, Subjective and Objective Sleep, Chronotype and Circadian Rhythms with Depressive Symptoms in Insomnia Disorder
Source: Brain Sci. 2023 Apr 4;13(4):613. doi: 10.3390/brainsci13040613 (PMC10136589; doi:10.3390/brainsci13040613)
Supplement: Supplementary file 1 [file brainsci-13-00613-s001.zip › brainsci-2232681-supplementary.pdf]

	Sleep quality	Circadian preference	Dysfunctional beliefs	Anxiety	TST$_d$	SOL$_d$	WASO$_d$	TST$_{psg}$	SOL$_{psg}$	WASO$_{psg}$	DLMO	Phase HBT	Sleep misperceptio
Depressive symptoms	0.298^	-0.306^	0.321^	0.607^	0.138	0.051	-0.042	-0.224*	0.055	-0.094	-0.073	0.106	0.021
Sleep quality	-	-0.166	0.258^	0.367^	-0.310^	0.338^	0.110	-0.274^	0.201*	0.158	-0.026	0.021	0.273*
Circadian preference		-	-0.272^	-0.145	-0.198*	-0.189*	0.146	0.088	-0.191*	0.033	-0.151	-0.089	0.119
Dysfunctional beliefs			-	0.233*	-0.046	-0.029	-0.002	0.126	0.009	0.014	0.095	-0.018	0.078
Anxiety				-	0.137	0.086	-0.022	-0.155	-0.029	-0.076	-0.188	0.163	0.032
TST$_d$					-	-0.226*	-0.430^	0.149	0.068	-0.201*	0.146	-0.173	-0.470^
SOL$_d$						-	-0.108	-0.095	-0.189*	-0.036	-0.131	0.102	0.243*
WASO$_d$							-	-0.175	-0.151	0.367^	-0.005	-0.126	0.464^
TST$_{psg}$								-	-0.254^	-0.350^	0.035	-0.070	-0.092
SOL$_{psg}$									-	0.003	0.165	0.031	-0.023
WASO$_{psg}$										-	-0.136	0.057	0.251^
DLMO											-	-0.796^	-0.158
Phase HBT												-	0.103
Sleep mispercepti													-

on

Table S1. Correlations between subjective and objective variables. Key: Depressive symptoms – PHQ-9; Sleep quality - PSQI; Circadian preference - CSM; Dysfunctional beliefs - DBAS; Anxiety - GAD-7; DLMO – dim light melatonin onset; TST – total sleep time$_d$ (diary); SOL – sleep onset latency$_d$ (diary); WASO$_d$ – wake after sleep onset (diary);); PSG – polysomnography; Phase HBT; habitual bedtime sleep diary-DLMO; * $p<0.05$; ^ $p<0.001$.
